# Systemic Administration of Substance P Recovers Beta Amyloid-Induced Cognitive Deficits in Rat: Involvement of Kv Potassium Channels

**DOI:** 10.1371/journal.pone.0078036

**Published:** 2013-11-12

**Authors:** Patrizia Campolongo, Patrizia Ratano, Maria Teresa Ciotti, Fulvio Florenzano, Stefania Lucia Nori, Roberta Marolda, Maura Palmery, Anna Maria Rinaldi, Cristina Zona, Roberta Possenti, Pietro Calissano, Cinzia Severini

**Affiliations:** 1 Department of Physiology and Pharmacology, Sapienza University of Rome, Rome, Italy; 2 Institute of Cell Biology and Neurobiology, CNR, Rome, Italy; 3 European Brain Research Institute, Rome, Italy; 4 Department of Medicine and Surgery, University of Salerno Medicine Campus, Baronissi (SA), Italy; 5 Department of Neuroscience, University of Rome “Tor Vergata”, Rome, Italy; 6 IRCCS Fondazione Santa Lucia, Rome, Italy; University of the Witwatersrand, South Africa

## Abstract

Reduced levels of Substance P (SP), an endogenous neuropeptide endowed with neuroprotective and anti-apoptotic properties, have been found in brain and spinal fluid of Alzheimer's disease (AD) patients. Potassium (K^+^) channel dysfunction is implicated in AD development and the amyloid-β (Aβ)-induced up-regulation of voltage-gated potassium channel subunits could be considered a significant step in Aβ brain toxicity. The aim of this study was to evaluate whether SP could reduce, *in vivo*, Aβ-induced overexpression of Kv subunits. Rats were intracerebroventricularly infused with amyloid-β 25–35 (Aβ_25–35,_ 20 µg) peptide. SP (50 µg/Kg, i.p.) was daily administered, for 7 days starting from the day of the surgery. Here we demonstrate that the Aβ infused rats showed impairment in cognitive performances in the Morris water maze task 4 weeks after Aβ_25–35_ infusion and that this impairing effect was prevented by SP administration.

Kv1.4, Kv2.1 and Kv4.2 subunit levels were quantified in hippocampus and in cerebral cortex by Western blot analysis and immunofluorescence. Interestingly, SP reduced Kv1.4 levels overexpressed by Aβ, both in hippocampus and cerebral cortex.

Our findings provide *in vivo* evidence for a neuroprotective activity of systemic administration of SP in a rat model of AD and suggest a possible mechanism underlying this effect.

## Introduction

Substance P (SP) is an 11-aa neuropeptide, member of the tachykinins family, widely distributed in the central nervous system (CNS). Several studies provided both *in vitro* and *in vivo* evidence of an involvement of the SP receptor (NK1) in neuronal development/survival, with SP exerting a trophic role and acting as a non-specific growth factor in both peripheral and central nervous tissues [1]–[3]. Reduced SP levels have been observed in cortical regions of post-mortem brain tissues [4]–[6] and in the cerebrospinal fluid [7] of patients suffering from Alzheimer's disease (AD), suggesting a significant involvement of SP in AD pathology [6], [8]. Though Aβ_1–42_ is considered the predominant pathological structure in AD [9], minor fragments have been identified; among them the highly toxic Aβ_25–35_ peptide represents the neurotoxic domain of the native, full-length peptide Aβ_1–42_ [10], [11]. The Aβ_25–35_ fragment is endogenously present in AD patients brain [11], [12] and can be produced by enzymatic cleavage of the full-length peptide Aβ [11], [13]. As recently reported, the endecapeptide Aβ_25–35_, which itself shows β-sheet structure [14], [15] is able to provoke long-lasting pathological alterations comparable to the human disease [16].

Literature data show that Aβ_25–35_-treated rodents develop behavioral impairments reminiscent of AD physiopathology [17], particularly spontaneous alternation, passive avoidance and water-maze learning deficits [16], [18], [19]. At CNS level, SP-immunoreactive cells are distributed in several brain regions implicated in the control of cognition and emotionality [20].

Evidences from literature suggest that SP facilitates cognitive functions when locally administered into particular brain regions or after systemic administration in rats [21]–[23]. Interestingly, consistent data indicate that SP plays a crucial role not only in memory formation and reinforcement but also in preventing brain aging-related memory decline [24], [25]. Kowall and co-workers [26] demonstrated that SP, co-administered together with beta-amyloid (Aβ) peptide into the rat cerebral cortex, prevented the amyloid-induced neuronal loss, which is considered one of the most important histopathological hallmark of AD.

Research on the mechanisms by which Aβ mediates neurotoxicity has made great strides over the last decade. Extensive growing evidence suggest that Aβ alters cellular homeostasis and neuronal signalling through several mechanisms crucially involving the potassium (K^+^) channels modulation [27]–[30]. An increased activity of plasma-membrane voltage-gated potassium (Kv) channels can induce cell death, suggesting that these channels are involved in the aetiology of Aβ-induced toxicity and neuronal death [31]–[33].

In cerebellar granule neurons the increase in I_kA_ current density induced by Aβ_1–40_ is due to the selective up-regulation of Kv4.2 and Kv4.3 α-subunits expression [34], while exposure of hippocampal neurons to Aβ_1–42_ leads to an increase in Kv3.4 protein expression [32]. These results highlight the crucial role played by selective voltage-dependent potassium channels in the aetiology of Aβ-induced toxicity, although the identity of the Kv subunits modulated by Aβ depends on different examined neurons. In addition to these *in vitro* evidence, Pan and co-workers [35] reported a significant increased expression of Kv2.1, Kv1.4 and Kv4.2 subunits after intracerebroventricular (i.c.v.) injection of Aβ_25–35_ in the rat hippocampus and cerebral cortex.

On the other hand, potassium channel abnormalities have been reported in both neural and peripheral tissues of AD patients. In particular, K^+^ channel dysfunction has been demonstrated in fibroblasts [36] and platelets [37] of AD patients and post-mortem studies showed alterations of K^+^ channel expression in the brain [38], [39]. Moreover, an aberrant glutamate-dependent modulation of Kv1.3 channels was recently demonstrated in T lymphocytes from AD patients [40].

Taken together, these findings demonstrate that alteration of Kv channel subunits expression and activity are involved in learning and memory dysfunction and in AD. We recently demonstrated that SP is able to significantly reduce *in vitro* the Aβ-induced over-expression of Kv subunits [41]. Based on these results, in the present study we investigated whether treatment with SP can help the recovery from memory dysfunction induced by i.c.v. infusion of Aβ_25–35_ in rats, and whether this potential protective effect could be related to the SP modulation of Kv channel subunits expression.

## Materials and Methods

### Chemicals

Aβ_25–35_, Aβ_35–25_ and the other reagents were purchased from Sigma-Aldrich (St. Louis, Missouri). 1.3 mg/ml Aβ peptide stock solutions were prepared in phosphate-buffered saline (PBS: 0.01 M NaH_2_PO_4_, 0.15 M NaCl, pH 7.4) and incubated at 37°C for 3 days before use. This procedure is known to produce insoluble precipitates and to facilitate markedly the appearance of learning deficits in several tasks [17], [18].

### Animals

Subjects. Male Sprague–Dawley rats (280–320 g at the time of surgery; Charles River Laboratories, Calco, Italy) were group housed and maintained in a temperature-controlled environment (20°C±1°C) under a 12-h light/12-h dark cycle (0700–1900 h lights on) with food and water available ad libitum.

### Ethics Statement

All procedures involving animal care or treatments were approved by the Italian Ministry of Health (Rome, Italy) and performed in compliance with the guidelines of the European Communities Council Directive of 24 November 1986 (86/609/EEC). All efforts were made to minimize animal suffering and to reduce the number of animals used.

### Surgery and infusion procedure

Rats (280–320 g) were anesthetized with sodium pentobarbital (50 mg/kg, i.p.) and given atropine sulfate (0.1 mg/kg, i.p.) to maintain breathing. The skull was positioned in a stereotaxic frame (Kopf Instruments, Tujunga, CA, USA), an incision was made along the midline, the scalp was retracted, and the area surrounding bregma was cleaned and dried. Infusions into the left lateral ventricle (coordinates; AP = −0.80 mm, ML = +2.0 mm, DV = −4.5 mm with respect to bregma) [42] were performed by using 30-gauge injection needles connected to 50-µL Hamilton microsyringes by polyethylene (PE-20) tubing. The Aβ_25–35_ (2 µg/µl; 10 µL injection volume) or its vehicle (PBS 10 µL injection volume) were infused at the rate of 0.37 µL/min by an automated syringe pump (KD Scientific, Holliston, MA, USA). The injection needles were retained within the target area for 60 s following infusion to maximize diffusion.

### Drug treatments

Rats infused with A*β*_25–35_ or its vehicle were randomly assigned to 2 experimental groups: Saline and SP. Both A*β*_25–35_ and vehicle group received intraperitoneal (i.p) administration of SP 50 µg/ml/Kg die or its vehicle (saline solution 0.9%) for 7 days starting from the day of the surgery.

### Morris Water Maze

#### Task procedures

Twenty eight days after surgery, the rats were handled 1 minute per day for 3 days before training on the MWM task. The water maze was a circular tank, 1.83 m in diameter and 0.58 m in height, filled with water (23–24°C) to a depth of 20 cm. A transparent Perspex platform (20–25 cm) was submerged 2.5 cm below the surface of the water in the northwest quadrant of the maze during training and could not be seen by the rats. The maze was located in a room containing several visual cues. The experiments were performed accordingly to the procedure previously described [43]–[45].

#### Spatial Training (Acquisition)

Day 1–3. Rats were given a daily training session of 4 trials (60 sec each one) for 3 consecutive. On each trial, the animal was placed in the tank facing the wall at one of the four designated start positions and allowed to escape onto the hidden platform. If an animal failed to find the platform within 60 seconds, it was manually guided to the platform. The rat was allowed to remain on the platform for 15 seconds and was then placed into a holding cage for 25 seconds until the start of the next trial. The time each animal spent to reach the platform was recorded as the escape latency.

#### Probe (Memory retention)

Day 4. The retention of the spatial training was assessed 24 hours after the last training session. Rats were returned to the water maze for a 60-second probe trial (in which the platform was removed) starting from a new position different from the starting points used during acquisition. The parameter measured from the probe trial was the time spent in the quadrant containing the platform during training (target quadrant).

#### Reversal learning

Day 5–8. Rats were given a daily training session of 5 trials (60 sec each one) for 4 consecutive days. The platform position was changed every day (day 5 north, day 6 northwest, day 7 northeast, day 8 northwest). On each trial, the animal was placed in the tank facing the wall at one of the four designated start positions and allowed to escape onto the hidden platform. If an animal failed to find the platform within 60 seconds, it was manually guided to the platform. The rat was allowed to remain on the platform for 10 seconds and was then placed into a holding cage for 15 seconds until the start of the next trial. The time each animal spent to reach the platform was recorded as the escape latency.

Behavioral data from the training, probe and reversal learning were acquired and analyzed using an automated video-tracking system (Smart, Panlab, Harvard Apparatus). The escape latency for the spatial training and reversal learning working memory and the amount of time rats spent in the target zone in the probe test were analyzed.

### Primary cultures

Hippocampal cultures were prepared from brain of embryos Sprague–Dawley rats (Charles River) at embryonic day 17–18 (E17/E18), as previously reported [46]. Briefly, hippocampus was dissected out in Hanks' balanced salt solution buffered with Hepes and dissociated via trypsin treatment. Cells were plated at 1×10^6^ cells on 3.5-cm dishes precoated with poly-l-lysine. After 2 days of culturing in neurobasal medium with B-27 supplement (0.5 mM l-glutamine, 1% antibiotic penicillin/streptomycin), half of the medium was changed every 3–4 days. All experimental treatments were performed on 12-day *in vitro* (DIV) cultures in Neurobasal +½ B27 fresh medium.

### Preparation of enriched membrane protein extracts and Western blot analysis

Rat cortex and hippocampus membranes were prepared by using a simplified version of protocols previously described [35]. All procedures were performed at 4°C, and all solutions contained a mixture of protease inhibitors (1 µg/ml leupeptin and 1× protease inhibitor mixture) to minimize proteolysis. By using a glass homogenizer, tissues were homogenized in a hypoosmotic buffer containing 50 mM Tris HCl (pH 7.4). Nuclei and debris were pelleted by centrifugation at 1,000 *g* for 10 min. The supernatant was then centrifuged at 100,000 *g* for 1 hr and the resulting pellet was resuspended in lysis buffer (1% NP40, 50 mM Tris-HCl (pH 8) and 0.5 M Na_2_EDTA. The protein concentration of the resulting enriched membranes was then determined by using Bio-Rad protein assay solution with bovine serum albumin (BSA) as a standard. Solubilized membranes were stored frozen at −80°C until use.

For Western blot analyses, equal amounts of proteins (50 µg/lane) were loaded and run on standard 4–12% sodium dodecyl sulfate (SDS)-polyacrylamide gels in MOPS electrophoresis buffer according to manufacturer protocols (NuPage). After staining with Ponceau S to verify uniformity of protein loads/transfer, the membranes were analyzed for immunoreactivity. Incubation with primary antibodies was performed overnight at 4°C (β-actin, Sigma 1∶5000, rabbit anti Kv1.4, Kv2.1, and Kv4.2, Sigma 1∶500). Incubation with secondary antibodies peroxidase-coupled anti-mouse or anti rabbit (Sigma) was performed for 1 h at room temperature. Immunoreactivity was developed by enhanced chemiluminescence (ECL system; Amersham, Arlington Heights, IL) and visualized by autoradiography. For analysis of the Western blotting data, densitometric analysis was performed using Scan Analysis software.

### Immunofluorescence

After behavioral analysis, rats were anaesthetized with sodium pentobarbital and intracardially perfused with a saline solution followed by a 4% paraformaldehyde solution in phosphate buffer saline (PBS). Brains were post-fixed for 24 hours, transferred in 30% sucrose/PBS solution at 4°C until it sank and sectioned at a sliding freezing microtome (Leica, Wetzlar, Germany). Forty micrometers coronal sections were collected in 0.05% sodium azide/PBS in a culture well and stored at 4°C until usage. Double immunofluorescence was performed by sections incubation in a mix solution of the following primary antibodies: mouse anti-NeuN (dilution 1∶100; Millipore) and rabbit anti-Kv1.4 (1∶100; Sigma) in PBS containing 0.3% Triton-X for 2 days at +4 C. After three washes, sections were incubated in a mix of the following secondary antibodies: Alexa Fluor 488 conjugated donkey anti-mouse IgG and Alexa-Fluor 555 conjugated donkey anti-rabbit IgG (dilution 1∶200; Molecular Probes, Eugene, OR, USA) for 2 hours at room temperature. The last step was three washes in PBS and 40 min incubation with the Hoescht solution (1 ng/ml, Molecular Probes, Invitrogen) for nuclei visualization. After that, sections were mounted on slides, air dried and coverslipped using gel mount (Biomeda Corp., Foster City, CA, USA).

Primary hippocampal neurons, after 48 hs experimental treatment, were washed in PBS and fixed in 4% paraformaldehyde (w/v in PBS) for 15 min at room temperature. Fixed cells were washed in PBS, pH 7.4, permeabilized using 0.1% Triton X100-Tris-HCl, pH7.4 for 5 min and then incubated with primary polyclonal antibodies raised against the anti-Kv1.4 (1∶200) subunit and mouse anti-NeuN (1∶500) in PBS at 4°C overnight. Cells were then washed in PBS and incubated with a Alexa Fluor 488 (1∶800) conjugated donkey anti-mouse IgG and Alexa-Fluor 555 conjugated donkey anti-rabbit IgG (1∶500) for 60 min at room temperature. Nuclei were stained with Hoechst for 5 min at room temperature.

Immunofluorescence was examined under a confocal laser scanning microscope (Leica SP5, Leica Microsystems, Wetzlar, Germany). Confocal acquisition settings were identical among the different experimental cases. For figure production, brightness and contrast of images were adjusted by applying the same values, and by taking care to leave a tissue fluorescence background for visual appreciation of the all fluorescence intensity features and to help comparison between the different experimental groups. For hippocampal cultures, neurons were selected and acquired by NeuN identification. Final figures were assembled by using Adobe Photoshop 7 and Adobe Illustrator 10. Boundaries and subdivisions of cortical and hippocampal structures were identified on the base of the Hoescht histofluorescence using a rat brain atlas (Paxinos). Image acquisitions were performed on frontal cortex (somatosensory area) and CA1 hippocampal region.

### Image analysis

Image analysis was performed by using Imaris Suite 7.4® (Bitplane A.G., Zurich, Switzerland) software (surface and spot modules) on six different images derived from each experimental group. Image analysis was performed under visual control to determine thresholds that subtracts background noise and takes into account cellular structures. During image processing, the images were compared with the original raw data to make sure that no structures seen in the original data series were introduced or that structures present in the original data series were not removed. To evaluate the cell bodies and neuropil areas, their relative fluorescence intensity, vesicles diameters and relative fluorescence intensity, images were zoomed and two different masks were manually drawn. The first mask type was drawn using the NeuN channel by considering only cells clearly displaying a nucleus to selectively identify neurons. The second type was drawn only on neuropil portions by taking care to exclude cell bodies. After which the Hoescht and NeuN channels were deleted and measures were performed.

### Statistical Analysis

Statistical analysis was performed using SPSS 11.0.0 for Windows (SPSS Inc., USA). All results are expressed as mean ± SEM, with n the number of independent experiments. Results obtained from behavioral studies were analyzed with one- or two-way analysis of variance (ANOVA) with repeated measures when appropriate. Post hoc comparisons were performed using Tukey's test. Data from Western blot analysis and immunofluorescence were performed by ANOVA, followed by Tukey's test for multiple comparisons. The significance level was set at p<0.05 (*) and p<0.01 (**).

## Results

### Morris Water Maze

**Figure 1:**
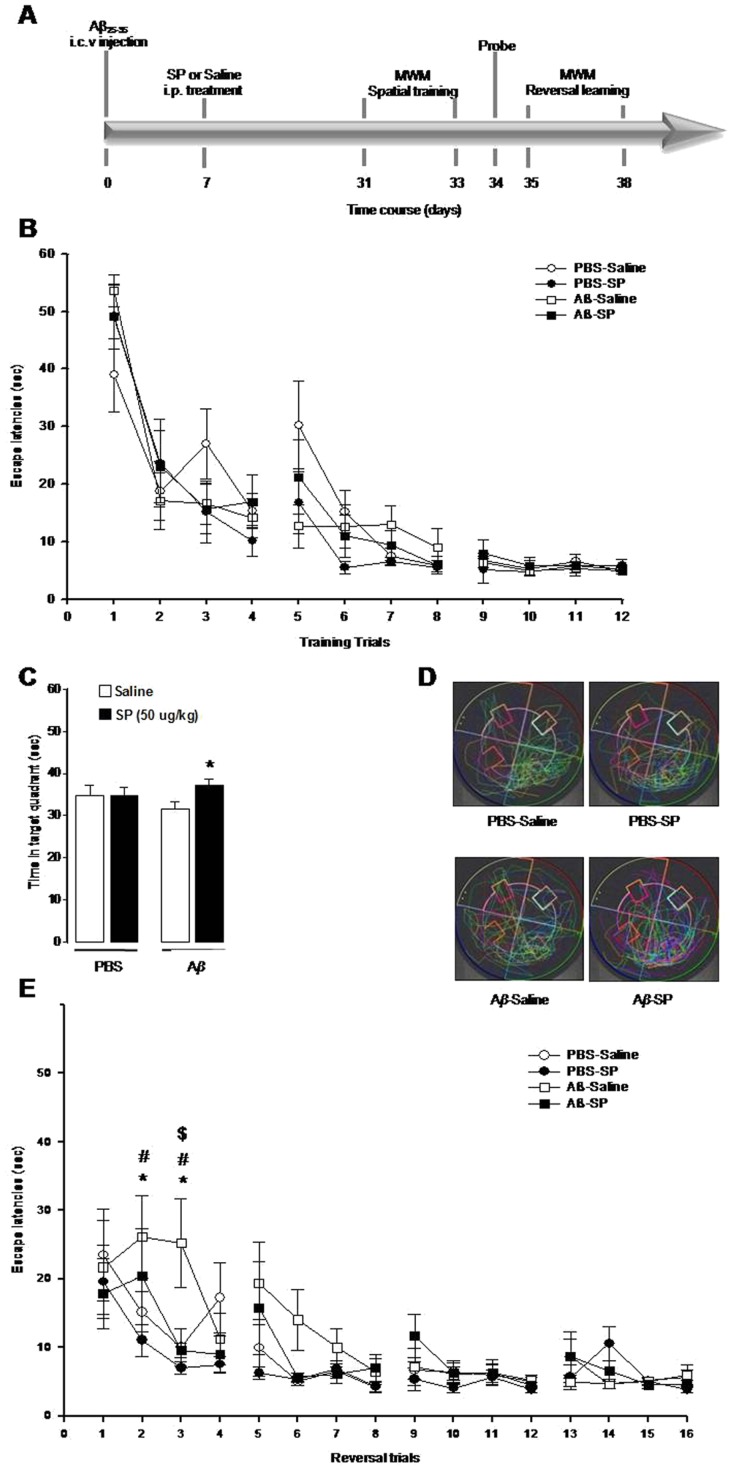
Neuroprotective effects of SP on memory impairments induced by intracerebroventricular injection of Aβ25–35. (a) Timeline and experimental design. All animals received an infusion (i.c.v.) of Aβ_25–35_ (2 µg/µl; 10 µL injection volume) or its vehicle (PBS 10 µL injection volume) and daily treated (7 days) with SP (50 µg/ml/Kg, i.p.) or its vehicle (saline solution 0.9%, i.p.). On the 31^st^ day after surgery rats were given a daily training session of 4 trials for 3 consecutive days (days 31^st^–33^rd^). On the 34^th^ day after surgery the retention of the spatial training was assessed during a 1 min probe trial. On the 35^th^ day after surgery rats were given a daily training session of 5 trials for 4 consecutive days (days 35^th^–38^th^). (b) Mean (±S.E.M.) distance traveled to the escape platform on 4 trials of 3 consecutive days of acquisition learning sessions. (c) Time spent (mean ±S.E.M.) during the 1-minute probe trial in the target quadrant and (d) illustrative paths of all animals for the probe test session. (e) Mean (±S.E.M.) distance traveled to the escape platform on 4 trials of 4 consecutive days of the reversal learning sessions (the hidden platform were relocated in a new position each day). * p<0.05 Aβ_25–35_/Sal *vs* PBS/Sal; # p<0.05 Aβ_25–35_/Sal *vs* PBS/SP; $ p<0.05 Aβ_25–35_/Sal *vs* Aβ_25–35_/SP. PBS/Sal, n = 10; PBS/SP, n = 10; Aβ_25–35_/Sal n = 12; Aβ_25–35_/SP, n = 10.

To examine whether treatment with SP can result in recovery from memory deficit induced by i.c.v. injection of Aβ_25–35_, the MWM task was carried out 4 weeks after Aβ_25–35_ inoculation (Figure 1a). Rats were early infused with A*β*_25–35_ or its vehicle and administered i.p. with SP or its vehicle for 7 days starting from the day of the surgery. After 4 weeks all groups were trained for 3 consecutive days on spatial training procedure of 4 trials session per day. During these sessions, rats had to learn to localize a hidden platform set always in a fixed place.

Figure 1b shows that all rats, regardless of any treatment, were equally able to acquire the cognitive task. Indeed, ANOVA for repeated measures (with trials as repeated measures) revealed a trial effect (*F*_(11,418)_ = 44.83; p<0.0001) but did not reveal any statistically significant interaction between trials and Aβ_25–35_ treatment (*F*_(11,410)_ = 1.06; p = 0.39) or an interaction between trials and SP (*F*_(11,418)_ = 0.81; p = 0.63) or among trials, Aβ_25–35_ and SP treatments (*F*_(11,418)_ = 1.53; p = 0.12). Moreover, ANOVA for repeated measures did not reveal a statistically significant Aβ_25–35_ (*F*_(1,38)_ = 0.15; p = 0.70) or SP (*F*_(1,38)_ = 0.65; p = 0.43) treatment effect or a statistically significant interaction between Aβ_25–35_ and SP treatments (*F*_(1,38)_ = 1.71; p = 0.20), thus highlighting that Aβ_25–35_ infusion did not induce any learning impairment since all rats were equally able to acquire the task.

The effect of Aβ_25–35_ inoculation on long-term memory was analyzed 24 h after the last acquisition day on a single 60 sec-probe trial (Figure 1c,d). During the probe test the platform was removed from the water maze tank and the time spent in the target quadrant where the platform was previously located was measured as a parameter of long-term memory retention. One-way ANOVA did not reveal any statistical significant Aβ_25–35_ (F_(1,38)_ = 0.045; P = 0.84), and SP (F_(1,38)_ = 2.28; p = 0.14) treatment effect, or a statistically significant interaction between Aβ_25–35_ and SP treatments (F_(1,38)_ = 2.26; p = 0.14). Post hoc comparisons performed on the interaction revealed a statistically significant difference between Aβ_25–35_/Sal and Aβ_25–35_/SP treated rats (p<0.05), thus showing that SP administration is able to reverse the impairing effects induced by Aβ on long-term memory retention.

Twenty-four hours after the probe test, rats were tested for reversal learning capabilities during a daily session of 4 trials for 4 consecutive days. The hidden platform were relocated in a new position every day, thus rats had to learn to quickly adjust their searching strategy.

Figure 1e shows the effect of SP administration on reversal learning performances. ANOVA for repeated measures revealed a trial effect (*F*_(15,570)_ = 11.77; p<0.0001) and a statistically significant interaction between trials and Aβ_25–35_ treatment (*F*_(15,570)_ = 2.03; p<0.012) but did not reveal any statistically significant interaction between trials and SP treatment (*F*_(15,570)_ = 1.29; p<0.20) or among trials, Aβ_25–35_ and SP treatments (*F*_(15,570)_ = 0.84; p<0.63). Post hoc comparisons revealed a statistically significant difference between Aβ_25–35_/Sal and PBS/Sal, Aβ_25–35_/Sal and PBS/SP and Aβ_25–35_/Sal and Aβ_25–35_/SP treated animals (p<0.05) in the second and/or third trial of the first day of reversal learning, thus showing that Aβ_25–35_ treated rats were impaired in the fast adjustment to a change in the platform location compared to the vehicle treated animals. Interestingly, we found that SP administration prevented the memory impairing effect induced by Aβ_25–35_. Moreover, ANOVA did not reveal a statistically significant Aβ_25–35_ treatment effect (*F*_(1,38)_ = 2.60; p = 0.11), SP treatment effect (*F*_(1,38)_ = 2.00; p = 0.17) or interaction between Aβ_25–35_ and SP treatments (*F*_(1,38)_ = 0.02; p = 0.87).

### Effect of SP on the Aβ-induced overexpression of Kv1.4 subunits in hippocampus and cerebral cortex

#### Western blot analysis

As reported by Pan et al. [35], infusion of aggregated Aβ_25–35_ in rat brain increased the expression of Kv1.4 and Kv2.1 in hippocampus and Kv4.2 in cerebral cortex. To examine whether the increased expression of these Kv channel proteins in Aβ_25–35_-treated rats could be modified by SP, Western blot studies were carried out on Kv2.1, Kv1.4, and Kv4.2 subunits, both in hippocampus and cerebral cortex.

**Figure 2:**
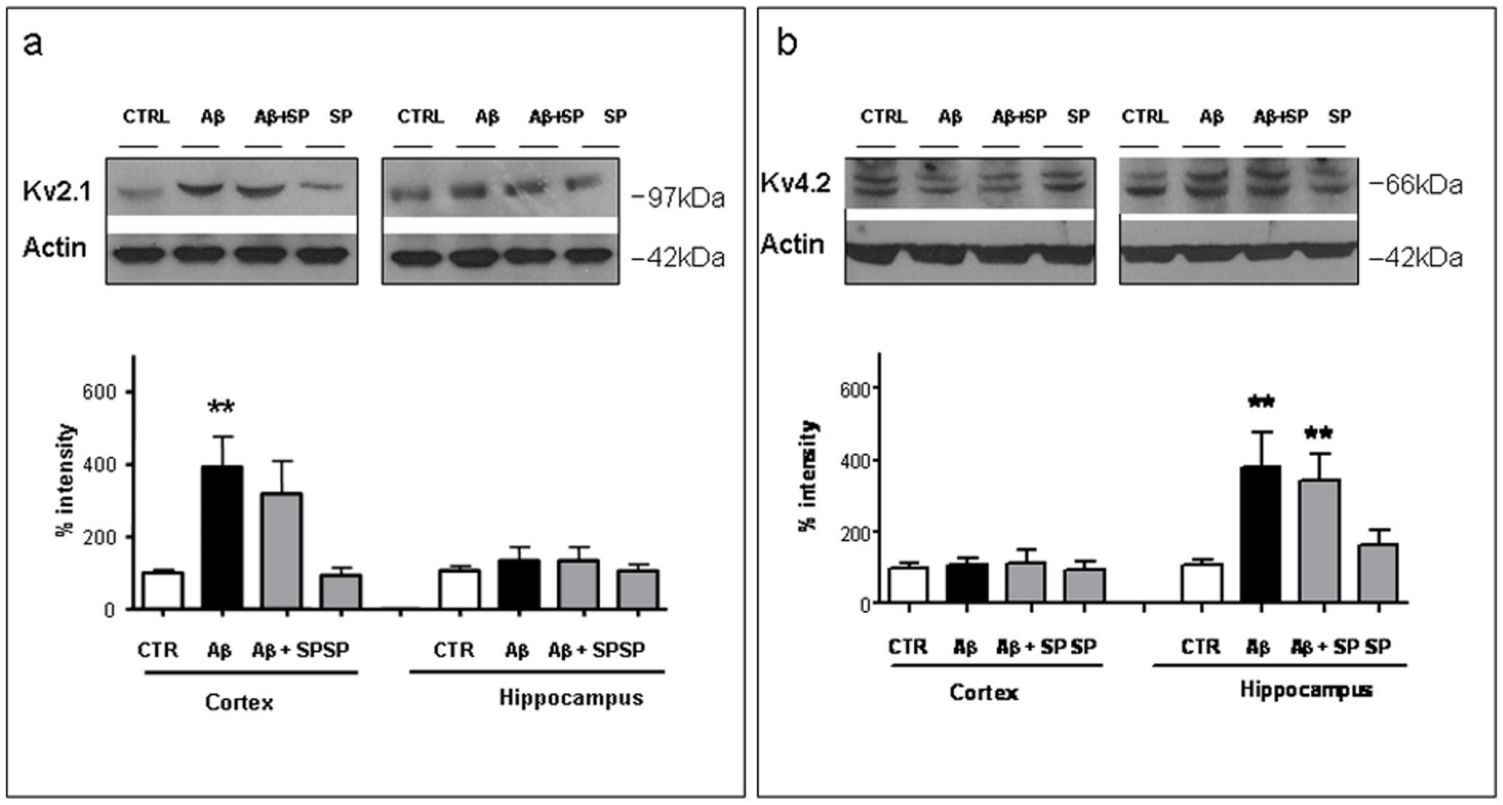
Western blot analysis of Kv 2.1 and Kv4.2 subunit expression in cerebral cortex and hippocampus. Representative immunoblot of cerebral cortex and hippocampus enriched membrane proteins (50 µg/lane) from (Ctr), Aβ_25–35_, Aβ_25–35_+SP and SP treated rats. Protein markers are shown at right (in kDa). The immunoreactive signals for **a**) Kv2.1 and **b**) Kv4.2 were quantified and normalized against β-actin and expressed as a percentage of control (CTR). Data represent mean (±SEM) from 3 independent experiments. Statistically significant differences were calculated by one-way analysis of variance (ANOVA) for repeated measures followed by Tukey's test for multiple comparisons (**p<0.01 versus Ctr value).

Results obtained by Western blot analysis confirmed the previously reported up-regulation of Kv1.4 and Kv2.1 in hippocampus and Kv4.2 in cerebral cortex. However, SP was able to modify only the increased expression of Kv1.4 protein, while SP treatment did not change Kv2.1 and Kv4.2 protein levels in both tissues (Fig. 2a and 2b).

**Figure 3:**
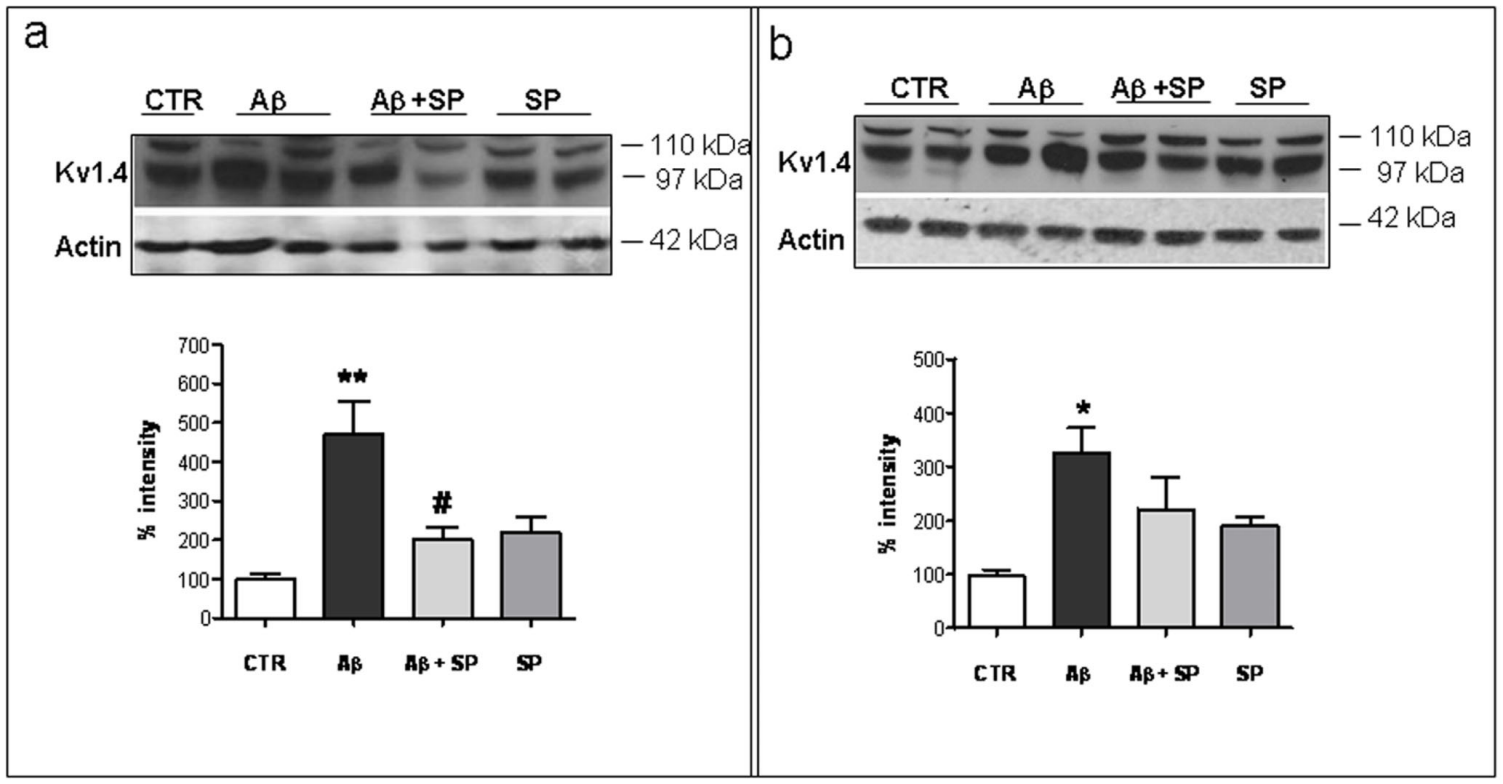
Western blot analysis of Kv1.4 subunit expression in hippocampus and cerebral cortex. Representative immunoblot of (**a**) hippocampus and (**b**) cerebral cortex enriched membrane proteins (50 µg/lane) from (Ctr), Aβ_25–35_, Aβ_25–35_+SP and SP treated rats. Protein markers are shown at right (in kDa). The immunoreactive signals at 97 and 110 kDa were quantified and normalized against β-actin and expressed as a percentage of the control (Ctr). Data represent mean (±SEM) from 5 independent experiments. Statistically significant differences were calculated by one-way analysis of variance (ANOVA) for repeated measures followed by Tukey's test for multiple comparisons (**p<0.01 versus Ctr value; #p<0.05 versus Aβ_25–35_ treatment).

Western blot analysis revealed that Kv1.4 protein was increased by about 5 times in hippocampus (Fig. 3a) and 3 times in cerebral cortex (Fig. 3b) of Aβ_25–35_-treated rats, confirming previous studies (Pan et al., 2004). As shown in Fig. 3a, SP treatment significantly reversed the Aβ_25–35_-dependent enhancement of Kv1.4 expression by about 2.5 times in hippocampus. In cerebral cortex of rats treated with Aβ_25–35_ and SP, however, we did not see a significant effect. Indeed, as shown in Fig. 3b, SP treatment slightly reversed the Aβ_25–35_-induced rise in Kv1.4 expression.

#### Immunofluorescence

**Figure 4:**
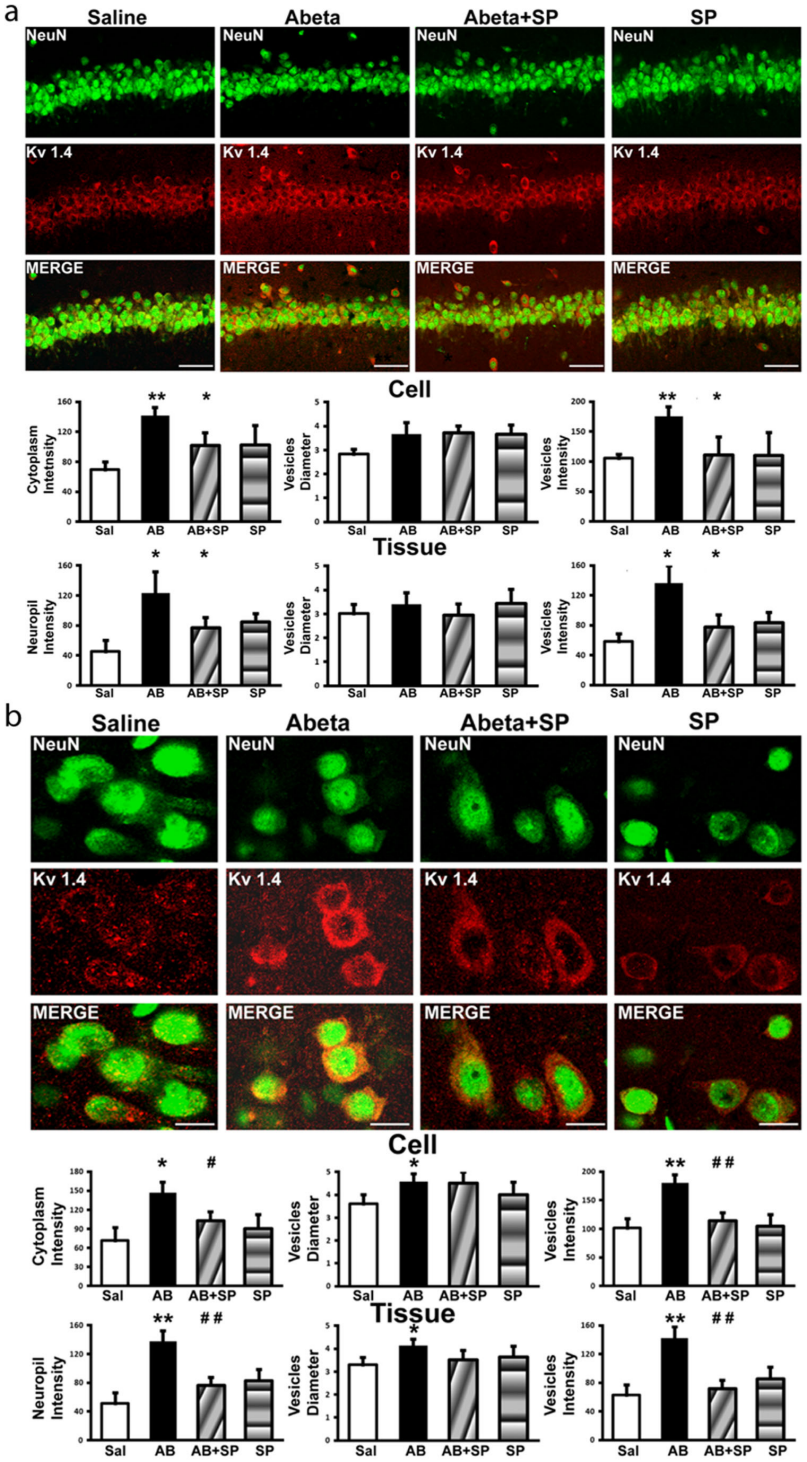
Immunofluorescence analysis of Kv1.4 subunit expression in hippocampus and cerebral cortex. Upper panel. Representative immunofluorescence photomicrographs showing Kv1.4 expression in **a**) hippocampus and **b**) frontal cortex after memory tests in the four experimental treatments: Control (Saline), Aβ_25–35_-i.c.v. treated rats (Abeta), Aβ_25–35_-i.c.v. and SP-i.p. treated rats (Abeta+SP), SP-i.p. treated rats (SP). Brain sections were labeled with the neuronal marker NeuN (green) and with the anti Kv1.4 antibody (red). As shown by the merge channel all neurons are Kv1.4 positive. Note the diffuse increase in Kv1.4 fluorescence intensity in the Abeta group and the decrease in the Abeta+SP group compared to the Control. Scale bar: a) 20 µm; b) 60 µm. Lower panel. Histograms showing image analysis performed on neuronal cytoplasm (first row) and the surrounding neuropil (second row). The indexes used were: total fluorescence intensity, vesicles diameters, and vesicles fluorescence intensity. Data represent means (±S.E.M.) obtained from three independent experiments. Statistically significant differences were calculated by one-way analysis of variance (ANOVA) for repeated measures followed by Tukey's test for multiple comparisons (**p<0.01 versus Saline; #p<0.05, ##p<0.01 versus Aβ_25–35_treatment).

Based on Western blotting indications, we decided to further investigate Kv1.4 tissue and cellular expression in hippocampus and cerebral cortex. As shown in figure 4a and 4b, in all treatment groups, both in hippocampus and cerebral cortex, Kv1.4 expression appeared mainly confined to neurons and surrounding neuropil inside vesicles and as background fluorescence. Interestingly, NeuN/Kv1.4 double immunofluorescence showed a virtually complete cellular co-expression of the two markers. Kv1.4 expression was selectively confined to the cell body and, in some cases, to the first portion of the dendritic tree with negligible, if any, nuclear staining (figure 4a and 4b). The neuropil appeared to have a high Kv1.4 expression although lower when compared to its expression in the cell body. Cerebral cortex and hippocampus apart, observations on other brain areas confirmed the widespread neuronal expression of the Kv1.4 through the brain: all the NeuN positive cells were Kv1.4 immunoreactive (data not shown). The intensity levels of Kv1.4 expression were increased in A*β*_25–35_-treated rats when compared to the other treatment groups (figure 4a and 4b). SP treatment significantly reversed the expression of Kv1.4 in both hippocampus and frontal cerebral cortex (somatosensory area) of A*β*_25–35_-treated rats.

Image analysis confirmed these qualitative observations (Figure 4a and 4b).

### Effect induced by SP on the Aβ-dependent overexpression of Kv1.4 subunits in hippocampal cultures

#### Western blot analysis

**Figure 5:**
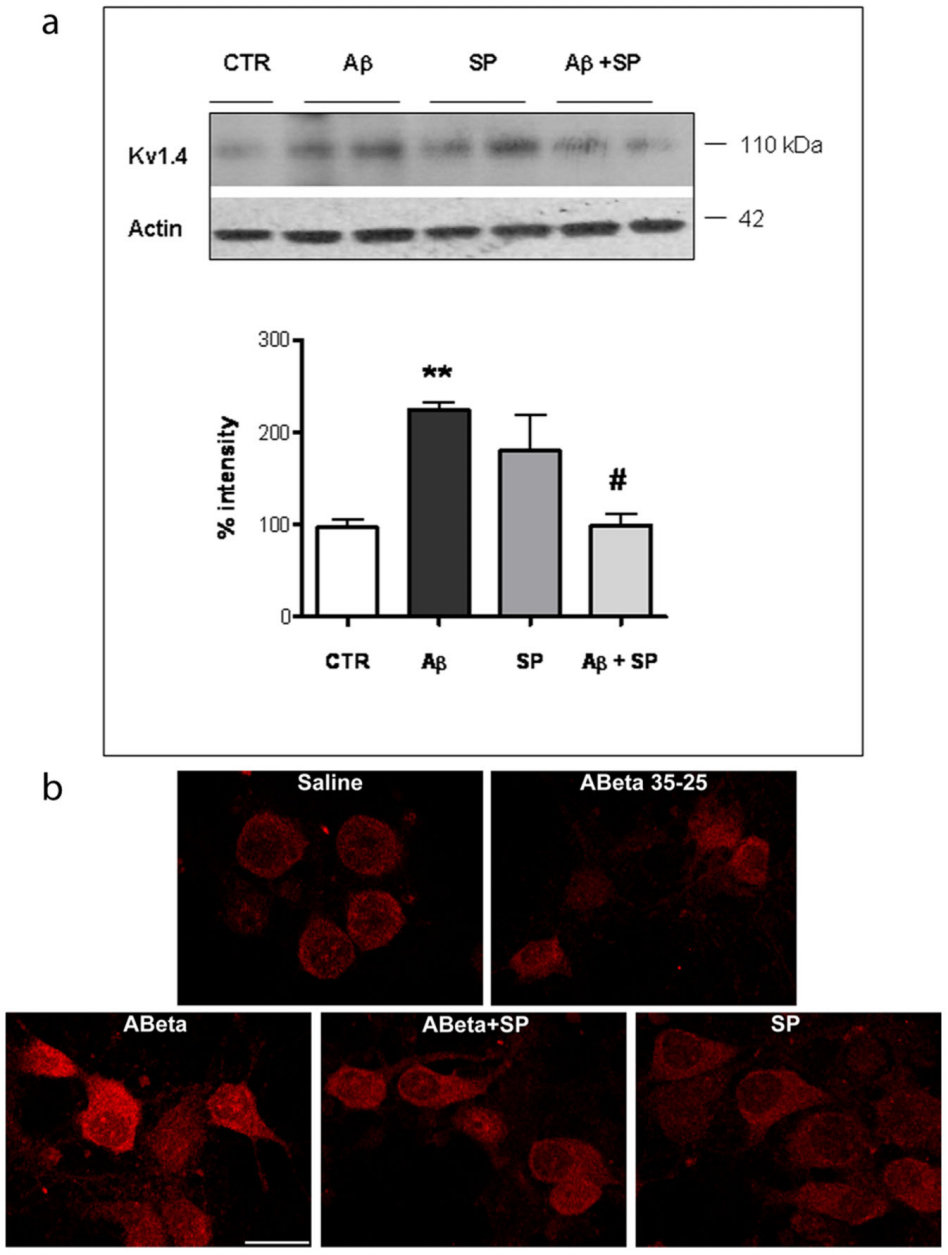
SP reduced Aβ25–35-induced overexpression of Kv1.4 subunit in rat hippocampal neurons. **a**) Example of Western blot obtained from hippocampal cultures exposed to 20 µM Aβ_25–35_ (Aβ alone or in the presence of SP (100 nM) and analyzed 48 h later using a polyclonal antibody against Kv1.4 subunit. The same blots were stripped and reprobed with an antibody against β-actin as internal control (lower panels). Quantitative analysis is depicted below the blots and was determined by band densitometry analysis considering the values found in CTR cells as 100. Data represent means (±S.E.M.) obtained from 4 independent experiments run in duplicate. (**p<0.001 versus CTR, #p<0.05 versus Aβ_25–35_ treatment). **b**) Representative immunofluorescence photomicrographs showing Kv1.4 expression in primary hippocampal cultures. Note the increase in immunofluorescence in the Aβ_25–35_ neurons, as compared to control neurons, reversed by SP treatment. Images were obtained from three independent experiments. Scale bar: 20 µm.

We previously demonstrated that Aβ_25–35_ in cultured cerebellar neurons caused an increase both in the I_kA_ and in the I_KV_ amplitude. We also found that SP suppressed the Aβ_25–35_-induced activation of both these currents, selectively reducing up-regulation of Kv4.2 and Kv4.3 channel subunits expression [41]. Since *in vivo* data indicated that SP treatment specifically modulates expression of Kv1.4 subunit in hippocampus, we tested whether the expression levels of Kv1.4 subunit could be modified in primary hippocampal cultures. Western blot analysis on hippocampal extracts treated with Aβ_25–35_ (20 µM) for 48 h revealed an increase of Kv1.4 subunit expression. As shown in Fig. 5a, SP (100 nM) prevented the Aβ_25–35_-induced overexpression of Kv1.4 subunits. In addition, some cells were treated with the reverse-sequence peptide Aβ_35-25_ (20 µM), but no differences in Kv1.4 subunit expression were observed between this peptide and control cultures (data not shown).

#### Immunofluorescence

Kv1.4 subunit expression was investigated by immunofluorescence in primary hippocampal cultures to extend the *in vivo* observations. Hippocampal neurons in control conditions or treated with the reverse-sequence peptide Aβ_35-25_ (20 µM) exhibited a low Kv1.4 immunofluorescence intensity level, mainly confined to vesicular structures residing in the cytoplasm and the most proximal neuritic portions (Fig. 5b). After 48 h incubation with Aβ_25–35_ (20 µM), several neurons displayed an increase in immunoreactivity, reversed by Aβ_25–35_ plus SP treatment. Importantly, treatment with SP alone did not trigger relevant variations in the Kv1.4 immunofluorescence intensity.

## Discussion

The present study provides the first *in vivo* evidence, to our knowledge, for a protective effect of SP against the cognitive impairment induced by infusion of Aβ_25–35_ in rats and it identifies Kv channel subunits as possible modulator of SP effects on memory rescue. To evaluate whether SP was able to prevent the cognitive dysfunction in a non-transgenic model of AD, we performed the MWM task, which is considered one of the best choice to investigate cognitive impairments in rodents. This task permits to evaluate the most affected cognitive processes in the onset symptoms associated with AD [47] and when based on a serial reversal procedure, is considered a useful tool to investigate episodic-like memory in rodents in respect to one-trial behavioral tasks [48]. In the present *in vivo* study, we found that all rats were equally able to learn and acquire the task and had similar motor and visual capabilities. However, we found that SP–treated rats show an ameliorated performance when long-term memory was tested, as highlighted by the fact that SP-treated rats remember better where the platform was during the training, since they spend more time swimming in the quadrant where the platform was (target quadrant) than Aβ_25–35_-infused rats. Interestingly, we found that Aβ_25–35_-injected rats present deficits in the reversal learning capabilities, which have been roughly compared to episodic-like memory in humans [48], [49]. Impairment of episodic memory has been found to be a marker for future development of AD based on convergent data from asymptomatic AD-related mutation carriers, longitudinal studies of patients with mild cognitive impairment, and epidemiological studies of incident AD cases [50], [51].

Notably, systemic SP treatment was able to improve long-term memory and reversal learning performances [20], [24], [52], [53], confirming the notion that SP easily crosses the blood-brain barrier [54].

The present findings confirm the emerging notion that specific memory deficits, rather than a general learning dysfunction, may occur in the early onset of AD. Previous studies in transgenic mice, indeed, report that deficits in spatial memory but not in learning capabilities early occur [55]–[57]. Although the mechanisms underlying this phenomenon have still to be clarified, taken together these results are consistent with human data, reporting that the main clinical symptom of AD is the early memory impairment, which then develops in dementia during the progression of the pathology [58], [59].

In the aetiology of Aβ neuronal death and AD, recent evidence points to an increase in voltage–gated potassium (Kv) channel current and to an up-regulation of selective voltage-gated potassium channels subunits, depending on different examined neurons [33]. In addition to the well known neuroprotective properties of SP, we recently demonstrated that SP was able to decrease Aβ_25–35_-induced neuronal death in rat cerebellar granule neurons through a selective regulation of the Kv4.2 and Kv4.3 channel subunits overexpressed by Aβ_25–35_ exposure [41]. We also demonstrated that, in the same neurons, SP stimulates non-amyloidogenic APP processing, thereby reducing the possibility of generation of toxic Aβ peptides in brain [60]. It thus appears that the toxic effect of Aβ and the neuroprotective effect of SP may be ascribed, at least in part, to their opposite actions on these currents. In support of this hypothesis is the evidence that i.c.v. injection of Aβ_25–35_ significantly impairs spatial memory of rats in the Morris water maze and increased the expression, both at mRNA and protein levels, of Kv2.1 and Kv1.4 in hippocampus and of Kv4.2 in cortex of Aβ-treated rats [35].

In the present study, we confirmed the up-regulation of Kv2.1, Kv1.4, and Kv4.2 subunits in Aβ treated rats.

However, SP was able to interfere only with Kv1.4 protein dysregulation. The diversity of Kv channels underlies much of the variability in the active properties between different mammalian central neurons [61]. The different structure of these proteins could be responsible for the selective modulation of Kv1.4 upregulation, without affecting Kv2.1 and Kv4.2 expression. In addition, the activity of Kv channels can be modulated indirectly *via* signal transduction pathways leading to modifications of Kv channel function and SP receptor activation involved different transduction pathways [62].

As revealed by Western blot analysis, SP significantly reduced, in the hippocampus, the over-expression of Kv1.4 subunits induced by Aβ treatment. In the whole cerebral cortex the mild Aβ-induced increase in Kv1.4 subunits was not significantly affected by SP treatment.

Interestingly, when specifically analyzing the tissue distribution of this protein in cerebral cortex by immunofluorescence, we found that in Aβ-treated rats, Kv1.4 expression level is increased in frontal cortex (somatosensory area), as compared to control rat brains. Notably, by immunofluorescence, we observed that SP was able to normalize the Kv1.4 subunits over-expression both in hippocampus and somatosensory cortex. The effect of SP on Kv1.4 subunit expression was further confirmed in cultured hippocampal neurons, indicating that SP is able to cross the blood-brain barrier and directly act on hippocampal neurons.

We suppose that the increased expression of Kv1.4, Kv2.1, and Kv4.2 we found in Aβ-treated rats could be at least partially responsible for the memory impairment detected in Aβ-treated rats. Increased K^+^ channel outward currents may indeed result in a decrease in neuronal excitability and K^+^ channels have been proved to be involved in the regulation of cognitive processes and altered in AD [38], [39]. Supporting our hypothesis, it has been demonstrated that i.c.v. injection of antisense oligodeoxyribonucleotide to Kv1.1, by reducing the expression of its particular intracellular mRNA target, provoked hippocampal-dependent memory loss in rat [63] and KV1.1 and KV1.3 channel blocker improves associative learning in rats [64].

On the whole, our results indicate that SP is able to modulate *in vivo* the expression of specific Kv channel subunits, known to be upregulated by Aβ treatment. The antiamnesic effect of SP shown in our rat model of AD could be of clinical relevance for a better understanding of AD development and it might represents a potential disease-modifying agent.

Since the targeted drug delivery to the central nervous system as treatment of neurodegenerative disorders such as AD, is restricted due to the limitations posed by the blood-brain barrier, the availability of a natural neuropeptide able to easily cross the blood-brain barrier may be a valuable therapeutic tool for AD treatment.
